# A Sister-Group Comparison of Branching and Pedicellariae in Brittlestars (Echinodermata: Ophiuroidea)

**DOI:** 10.1093/iob/obad013

**Published:** 2023-05-03

**Authors:** R L Turner, B O O'Neill

**Affiliations:** Department of Ocean Engineering and Marine Sciences, Florida Institute of Technology, Melbourne, FL 32901, USA; Department of Ocean Engineering and Marine Sciences, Florida Institute of Technology, Melbourne, FL 32901, USA; 11125 West 45th Ave., Wheat Ridge, CO 80033, USA

## Abstract

Branching of arms and presence of pedicellariae are characters among ophiuroids found only in the order Euryalida (snakestars and basketstars). Family Asteronychidae has neither character; family Euryalidae has 2 small clades with branched arms; and family Gorgonocephalidae has all species with pedicellariae and 3 or 4 clades with branched arms. Despite the rare occurrence of these characters in the Ophiuroidea, they might be key adaptations within the Euryalida that have led to relatively high diversification. Sister-group comparison of the distribution of these 2 characters among taxa indicates that neither character alone explains diversity patterns within the order. In particular, branching restricted to the tips of arms seems not strongly adaptive, probably for the lack of integration of basal forks with the disc. On the other hand, 2 clades of gorgonocephalids with basal branching exceed their snakestar sister groups in numbers of species, indicating an advantage of branching within the family. Unfortunately, the analysis cannot benefit from statistics, for at least 5 independent comparisons are required for a one-tailed sign test. Because branching and pedicellariae are probably not independent variables, future sister-group comparisons should be done only within the Gorgonocephalidae once clade structure is better clarified with increased taxon sampling (10 currently missing genera) and resolution of intra-generic inconsistencies in the most recent cladograms available. Branching might confer upon gorgonocephalid basketstars a more efficient use of pedicellariae for upstream capture of zooplankton over their snakestar relatives as well as over the Euryalidae, which retain ancestral downstream capture by mucus-laden podia.

## Abstract (es)

German

Die Verzweigung der Arme und das Vorhandensein von Pedicellarien sind Merkmale unter Ophiuroiden, die nur in der Ordnung Euryalida (Schlangensterne mit unverzweigten Armen und Korbsterne mit verzweigten Armen) vorkommen. Die Familie Asteronychidae hat kein Merkmal; die Familie Euryalidae hat zwei kleine Kladen mit verzweigten Armen; und die Familie Gorgonocephalidae hat alle Arten mit Pedicellarien und drei oder vier Kladen mit verzweigten Armen. Trotz des seltenen Vorkommens dieser Merkmale in den Ophiuroidea könnten sie Schlüsselanpassungen innerhalb der Euryalida sein, die zu einer relativ hohen Diversifizierung geführt haben. Eine Schwestergruppenanalyse der Verteilung dieser beiden Merkmale unter den Taxa zeigt, dass keines der Merkmale allein die Diversitätsmuster innerhalb der Ordnung erklärt. Insbesondere die auf die Armspitzen beschränkte Verzweigung scheint nicht stark adaptiv zu sein, wahrscheinlich wegen fehlender Integration von Basalgabeln in die Scheibe. Andererseits übertreffen zwei Kladen von Gorgonocephaliden mit basaler Verzweigung ihre Schlangenstern-Schwestergruppen in der Anzahl der Arten, was auf einen Vorteil der Verzweigung innerhalb der Familie hinweist. Leider kann die Analyse nicht von Statistiken profitieren, da für einen einseitigen Vorzeichentest mindestens fünf unabhängige Vergleiche erforderlich sind. Da Verzweigung und Pedicellariae wahrscheinlich keine unabhängigen Variablen sind, sollten zukünftige Schwestergruppenvergleiche nur innerhalb der Gorgonocephalidae durchgeführt werden, sobald die Klade-Struktur durch vermehrte Taxon-Stichproben (10 derzeit fehlende Gattungen) und durch Auflösung von Inkonsistenzen innerhalb der Gattungen in den neuesten verfügbaren Kladogrammen besser aufgeklärt ist. Die Verzweigung könnte gorgonocephaliden Korbsternen eine effizientere Nutzung von Pedicellarien für den stromaufwärts gelegenen Fang von Zooplankton gegenüber ihren Schlangenstern-Verwandten sowie gegenüber den Euryalidae verleihen, die den stromabwärts gelegenen Fang durch schleimbeladene Füsschen der Vorfahren beibehalten.

## Introduction

**Fig. 1:**
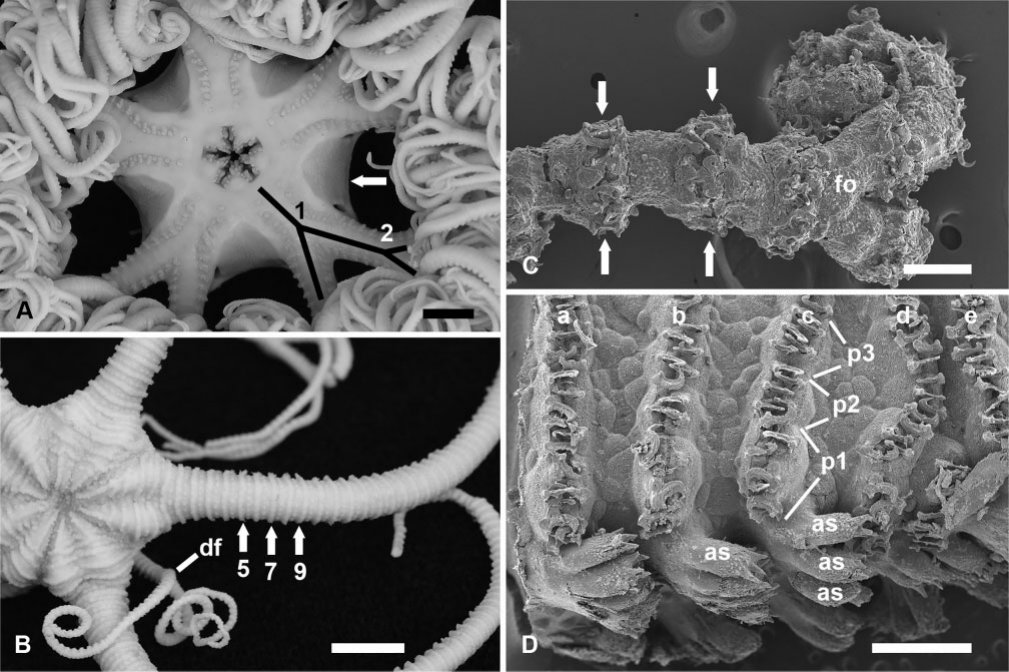
Branching and pedicellariae of the Euryalida. A. *Gorgonocephalus eucnemis*, oral view, five highly branched rays with first fork (1) within the margin of the disc (arrow). The second fork (2) is outside the disc margin. Black lines trace the basal branching of this species. Scale bar, 1 cm. B. *Ophiocnida isidis*, dorsal view, with five long arms that branch near the arm tips, giving an initial false impression of a snakestar. In this image, one arm remains unbranched for at least 36 segments before going out of view; segments 5, 7, and 9 free of the disc are labeled; and bands of pedicellariae are indicated at the arrows. The first fork (df) of this distally branching species occurs far out on an arm. Scale bar, 1 cm. C. *Astrophyton muricatum*, distalmost fork (fo) near the arm tip with bands of pedicellariae spanning between the arrows. Scale bar, 200 μm. D. *Asteroporpa annulata*, lateral view of arm with five bands (a–e) of pedicellariae above ventrolateral rows of arm spines (as). Three (p1–p3) of several pedicellariae of band c are labeled, each bearing five to six valves. Scale bar 1 mm.

Although echinoderms are generally viewed as animals with body plans arranged in five rays, axes, or ambulacra, there are groups that have more than five rays. Addition of rays occurs by insertion of new rays between or among the original five rays from the ring canal in some seastars (Asteroidea; Lawrence and Komatsu 1990) and some brittlestars (Ophiuroidea; Okanishi and Mah 2020). The addition of rays increases the length of usable ambulacra and the podia (tube feet) that line them to magnify the functions of podia for feeding, locomotion, gas exchange, or other roles (Lawrence 2012). Alternatively, other echinoderms increase the length of usable ambulacra by branching of the original five rays. Branching occurs only in suspension-feeders: many feather stars and sea lilies (Crinoidea; Messing et al. 2021) and some ophiuroids (Lawrence 2012). The ophiuroids with branching rays (or “arms” in ophiuroids) are found exclusively in the order Euryalida, a monophyletic taxon (Okanishi and Fujita 2013; O'Hara et al. 2014, 2018; Thuy and Stöhr 2016). Those euryalids with branching arms are called “basketstars” (Fig. 1A), and those with simple (non-branching) arms are called “snakestars” (Fig. 1B); these terms refer to body morphology and are not taxa. The Euryalida includes three families: Asteronychidae, Euryalidae, and Gorgonocephalidae, each with different proportions of basketstar and snakestar species.

Most taxonomic treatments of the Euryalida include descriptions of forks and branches of basketstars, but they rarely describe morphological and phylogenetic patterns. The earliest observation of branching in the Euryalida was by Winthrop (1670) on *Piscis Echino-stellaris Visciformis* (currently *Gorgonocephalus arcticus*) more than 350 years ago. Having counted some of the branches and the forks at which they are joined on his specimen, Winthrop estimated the total number of terminal branches on a specimen to be 81,920, a number found later to be spuriously high by Lyman (1877). More significantly, Winthrop noted that the branch lengths between forks were unequal and that the branching pattern was asymmetric. Lyman (1877), who wrote the next major treatment of branching, delved further into the patterns of asymmetry and number of forks for four species of basketstar in the Euryalidae and Gorgonocephalidae. Lyman noted that the number of segments between forks must be fixed as the animal grows; even then echinoderm biologists knew that addition of ambulacral ossicles is subterminal or penultimate, a condition now called the Ocular Plate Rule (Mooi et al. 1994). Döderlein (1912), in his proposal on phylogeny of the Euryalida, observed that the genera and species of gorgonocephalid are considerably different in the formation of their arms and made the distinction between locomotor and feeding arms. Fedotov (1926) described the progression of branching in young growth stages of *G. eucnemis*, demonstrating the gradual growth of the disc almost to cover the first fork. Only more recently (Strathmann 1975; Lawrence 2012) has branching been viewed in more functional and theoretical contexts. The morphological, histological, and genetic foundations for the proclivity to branch in the Euryalida, and not to branch in the other ophiuroids, has not been addressed.

Another unique feature of some members of the Euryalida is the gorgonocephalous pedicellaria. Pedicellariae, specialized grasping appendages derived from spines, have long been known to occur in all sea urchins and their allies (Echinoidea) and in some groups of asteroids. Pedicellariae are now recognized to occur in one family of the Euryalida, the family Gorgonocephalidae, in which they serve primarily or exclusively for prey capture (Turner et al. 2021). Their presence as a distinguishing character of the family has been known for more than 100 years. The pedicellariae are arranged in raised bands around the arms of gorgonocephalids, often giving the arms an annulated appearance (Fig. 1B–D). The presence of pedicellariae in gorgonocephalids and absence from all other ophiuroids can be taken to indicate that pedicellariae are an autapomorphy of the family. Turner et al. (2021) documented the history of studies on gorgonocephalous pedicellariae, and there has been no treatment of what impact the presence of this character has had on diversification in the family.

Not all Euryalida are basketstars, and not all have pedicellariae. All gorgonocephalids have pedicellariae, but not all are basketstars. If no other ophiuroids have branching arms and pedicellariae, then we wondered in what way these two aggregate, categorical, and presumably heritable traits might be key innovations. De Queiroz (1998) wrote, “The idea that a particular trait can increase the diversification rate of a group has a long tradition in evolutionary biology.” Here, we describe branching patterns in arms of the Euryalida and compare sister groups (Barraclough et al. 1998) to see what might be revealed by the distribution of branching and pedicellariae and the relative taxonomic diversity within this order of the Ophiuroidea.

## Material and methods

World Register of Marine Species (Stöhr et al. 2022) was used as the authority for taxonomy. The order–family pair Euryalida and Euryalidae are problematic for the use of convenient common nouns and adjectives for scientific taxa. Going forward, we will use the terms “euryalidan” in reference to the Euryalida and “euryalid” for the Euryalidae.

Information was extracted from literature on the number of arms and whether arms were simple (unbranched) or branched, how far along the arm the first fork (Fig. 1A–B) occurred, and the maximal times that an arm forked. We recorded the number of arm segments to the first fork in all specimens, but noted the number of forks within the disc margin only for the largest specimens described in the literature: Whereas disc size and inclusion of forks increase during growth, the number of segments is constant because segments are added only at the arm tips (Lyman 1877; Mooi et al. 1994). As much as possible, data were taken from the original description of the taxon and confirmed by later work. Photographs and drawings were consulted if data were not available in the text. Because the presence of branching and multiple (>5) arms are of considerable interest among echinoderm systematists, failure to mention or illustrate these unique conditions in the description of a species was taken for the presence of five simple arms.

**Fig. 2:**
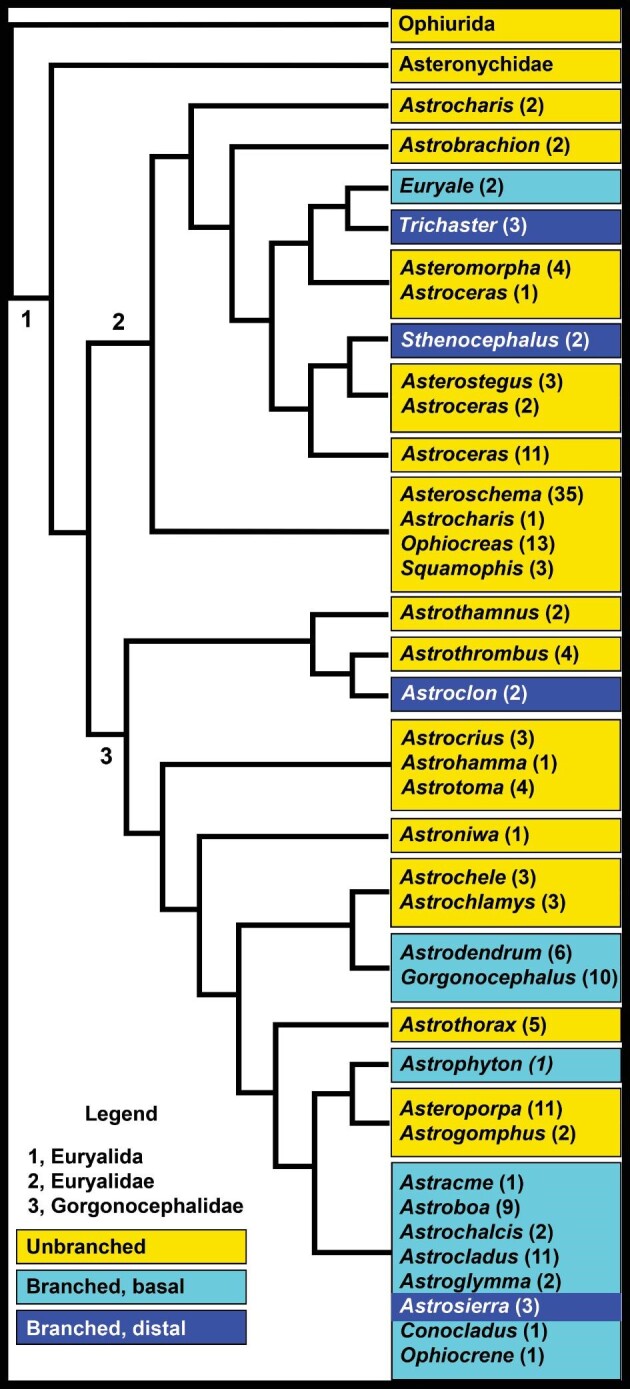
Simplified cladogram of Euryalida based on Okanishi and Fujita (2013, 2018), Okanishi et al. (2020), and Christodoulou et al. (2019b). Ophiurida is the outgroup. See Table 1 for species included in basketstar clades and their sister snakestar clades. Branches of the cladogram only indicate relationships, not mutation rate or geological time scale.

For comparison of sister groups, two cladograms were combined, although not without difficulty: The cladogram of Okanishi and Fujita (2013; their Fig. 1) with modifications from Okanishi and Fujita (2018) and Okanishi et al. (2020); the cladogram of Christodoulou et al. (2019b), which accompanies their paper (Christodoulou et al. 2019a). Neither cladogram included all euryalidan genera, nor were the same subsets of species used by the authors for each genus. Overlap of included taxa was fairly high between the two cladograms (62% of genera, 30% of species). In cases of disagreement, deference generally was given to the more recent cladogram (Christodoulou et al. 2019b) with a higher level of genetic sampling. Specific cases of troublesome disparities are described below. Our updated and simplified version is given in Fig. 2, and details for sister groups that we compare here are given in Table 1. We emphasize that Fig. 2 does not reflect an analysis of the original genetic data; it only serves to identify sister groups with contrasting body plans based on the presence and absence of branched arms and pedicellariae.

**Table 1:** Taxa in clades of Fig. 2. Clades are listed in their order, top to bottom, in Fig. 2, with the clade named after the first genus given in the colored boxed clade. Species in bolded text are those included in the cladograms of Okanishi and Fujita (2013, 2018), Okanishi et al. (2020), and Christodoulou et al. (2019b). Genera and species in clades of basketstars and their sister groups are listed; clades not used for comparison (snakestars with no sister group of basketstars) have only numbers of included genera and species. Genera and their species not represented in cladograms are listed here as “incertae sedis.” Presence (+) and absence (−) of basal (Fig. 1A) and distal (Fig. 1B) branching patterns and of pedicellariae are given.

			Branching pattern		
Clade name	Genera	Species	Basal	Distal	Pedicellariae
Ophiurida	46	404	−	−	−
		Family Asteronychidae			
Asteronychidae	4	12	−	−	−
		Family Euryalidae			
*Astrocharis*	1	2	−	−	−
*Astrobrachion*	1	2	−	−	−
*Euryale*	*Euryale*	***E. aspera* Lamarck, 1816**	+	−	−
		*E. purpurea* Mortensen, 1934	+	−	−
*Trichaster*	*Trichaster*	***T. acanthifer* Döderlein, 1927**	−	+	−
		*T. flagellifer* von Martens, 1866	−	+	−
		***T. palmiferus* (Lamarck, 1816)**	−	+	−
*Asteromorpha*	*Asteromorpha*	***A. capensis* (Mortensen, 1925)** **^1^**	−	−	−
		***A. koehleri* (Döderlein, 1898)**	−	−	−
		***A. rousseaui* (Michelin, 1862)**	−	−	−
		*A. tenax* Baker, 1980	−	−	−
	*Astroceras*	***A. kermadecensis* Baker, 1980**	−	−	−
*Sthenocephalus*	*Sthenocephalus*	***S. anopla* (H. L. Clark, 1911)**	−	+	−
		***S. indicus* Koehler, 1898**	−	+	−
*Asterostegus*	*Asterostegus*	***A. maini* McKnight, 2003**	−	−	−
		***A. sabineae* Okanishi and Fujita, 2014** ^3^	−	−	−
		***A. tuberculatus* Mortensen, 1933**	−	−	−
	*Astroceras*	***A. nodosum* Koehler, 1930**	−	−	−
		***A. spinigerum* Mortensen, 1933**	−	−	−
*Astroceras*	1	11	−	−	−
*Asteroschema* ^2^	4	52	−	−	−
		Family Gorgonocephalidae			
*Astrothamnus*	1	2	−	−	+
*Astrothrombus*	*Astrothrombus*	***A. chrysanthi* Matsumoto, 1918**	−	−	+
		***A. rigens* (Koehler, 1910)**	−	−	+
		***A. rugosus* H. L. Clark, 1909**	−	−	+
		***A. vecors* (Koehler, 1904)**	−	−	+
*Astroclon*	*Astroclon*	***A. propugnatoris* Lyman, 1879**	−	+	+
		***A. suensoni* Mortensen, 1911**	−	+	+
*Astrocrius*	3	8	−	−	+
*Astroniwa*	1	1	−	−	+
*Astrochele*	*Astrochele*	*A. laevis* H. L. Clark, 1911	−	−	+
		***A. lymani* Verrill, 1878**	−	−	+
		***A. pacifica* Mortensen, 1933**	−	−	+
	*Astrochlamys*	***A. bruneus* Koehler, 1912**	−	−	+
		***A. sol* Mortensen, 1936**	−	−	+
		*A. timoharai* Okanishi and Mah, 2020	−	−	+
*Astrodendrum*	*Astrodendrum*	*A. capense* (Mortensen, 1933)	+	−	+
		*A. elingamita* Baker, 1974	+	−	+
		*A. galapagense* A. H. Clark, 1916	+	−	+
		*A. laevigatum* (Koehler, 1897)	+	−	+
		***A. sagaminum* (Döderlein, 1902)**	+	−	+
		*A. spinulosum* Okanishi and Fujita, 2018	+	−	+
	*Gorgonocephalus*	*G. arcticus* Leach, 1819	+	−	+
		*G. caputmedusae* (Linnaeus, 1758)	+	−	+
		***G. chilensis* (Philippi, 1858)**	+	−	+
		*G. diomedeae* Lütken and Mortensen, 1899	+	−	+
		*G. dolichodactylus* Döderlein, 1911	+	−	+
		***G. eucnemis* (Müller and Troschel, 1842)**	+	−	+
		*G. lamarckii* (Müller and Troschel, 1842)	+	−	+
		***G. pustulatum* (H. L. Clark, 1916**)	+	−	+
		***G. sundanus* Döderlein, 1927**	+	−	+
		***G. tuberosus* Döderlein, 1902**	+	−	+
*Astrothorax*	1	5	−	−	+
*Astrophyton*		***Astrophyton muricatum* (Lamarck, 1816)**	+	−	+
*Asteroporpa*	*Asteroporpa*	***A. annulata* Örsted and Lütken in Lütken, 1856**	−	−	+
		***A. australiensis* H. L. Clark, 1909**	−	−	+
		*A. bellator* (Koehler, 1904)	−	−	+
		***A. hadracantha* H. L. Clark, 1911**	−	−	+
		***A. indicus* Baker, 1980**	−	−	+
		*A. koyoae* Okanishi and Fujita, 2011	−	−	+
		*A. lindneri* A. H. Clark, 1948	−	−	+
		***A. muricatopatella* Okanishi and Fujita, 2011**	−	−	+
		*A. paucidens* (Mortensen, 1933)	−	−	+
		*A. pulchra* H. L. Clark, 1915	−	−	+
		***A. reticulata* Baker, 1980**	−	−	+
	*Astrogomphus*	*Astrogomphus rudis* Verrill, 1899	−	−	+
		***Astrogomphus vallatus* Lyman, 1869**	−	−	+
*Astracme*	*Astracme*	***Astracme mucronata* (Lyman, 1869)**	+	−	+
	*Astroboa*	*A. albatrossi* Döderlein, 1927	+	−	+
		***A. arctos* Matsumoto, 1915**	+	−	+
		*A. clavata* (Lyman, 1861)	+	−	+
		*A. ernae* Döderlein, 1911	+	−	+
		***A. globifera* (Döderlein, 1902)**	+	−	+
		*A. granulatus* (H. L. Clark, 1938)	+	−	+
		***A. nigrofurcata* Döderlein, 1927**	+	−	+
		***A. nuda* (Lyman, 1874)**	+	−	+
		*A. tuberculosa* Koehler, 1930	+	−	+
	*Astrochalcis*	*Astrochalcis micropus* Mortensen, 1912	+	−	+
		***Astrochalcis tuberculosus* Koehler, 1905**	+	−	+
	*Astrocladus*	*A. africanus* Mortensen, 1933	+	−	+
		*A. annulatus* (Matsumoto, 1912)	+	−	+
		***A. coniferus* (Döderlein, 1902)**	+	−	+
		***A. dofleini* Döderlein, 1910**	+	−	+
		***A. euryale* (Retzius, 1783)**	+	−	+
		***A. exiguus* (Lamarck, 1816)**	+	−	+
		*A. goodingi* Baker, Okanishi, and Pawson, 2018	+	−	+
		***A. hirtus* Mortensen, 1933**	+	−	+
		*A. ludwigi* (Döderlein, 1896)	+	−	+
		*A. socotrana* Baker, Okanishi, and Pawson, 2018	+	−	+
		*A. tonganus* Döderlein, 1911	+	−	+
	*Astroglymma*	***A. sculptum* (Döderlein, 1896)**	+	−	+
		*A. spinosum* Mortensen, 1933	+	−	+
	*Astrosierra*	***A. amblyconus* (H. L. Clark, 1909)**	−	+	+
		*A. densus* Baker, 1980	−	+	+
		***A. microconus* (H. L.** Clark, 1914)	−	+	+
	*Conocladus*	***C. australis* (Verrill, 1876)**	+	−	+
	*Ophiocrene*	***O. aenigma* Bell, 1894**	+	−	+
Family Gorgonocephalidae incertae sedis					
		*Astrocaneum herrerai* (A. H. Clark, 1919)	+	−	+
		*Astrocaneum spinosum* (Lyman, 1875)	+	−	+
		*Astrocnida isidis* (Duchassaing, 1850)	−	+	+
		*Astrocyclus caecilia* (Lütken, 1856)	+	−	+
		*Astrocyclus somaliensis* Baker, Okanishi, and Pawson, 2018	+	−	+
		*Astrodictyum panamense* (Verrill, 1867)	+	−	+
		*Astrogordius cacaoticus* (Lyman, 1874)	+	−	+
		*Astroplegma expansum* Döderlein, 1927	+	−	+
		*Astrospartus mediterraneus* (Risso, 1826)	+	−	+
		*Astrozona munita* (Koehler, 1904)	−	−	+
		*Ophiozeta turgida* Koehler, 1930	−	−	+
		*Schizostella bifurcata* A. H. Clark, 1952	−	+	+

1 Treated as *Asteroschema capense* by Okanishi and Fujita (2013).

2 Inclusive genera and species divided into families Astrocharidae and Asteroschematidae by Okanishi and Fujita (2013) but considered in Euryalidae by Stöhr et al. (2022).

3 Possibly *Asterostegus* sp. of Okanishi and Fujita (2013).

All species of Euryalidae were included because at least one species of each of the 11 genera was represented in the cladogram of Okanishi and Fujita (2013); 8 of the 11 euryalid genera were included by Christodoulou et al. (2019b). We positioned *Asterostegus, Squamophis*, and *Trichaster* per Okanishi and Fujita (2013). This allowed addition of 38 species to our analysis of the Euryalidae. Inconsistencies in the distribution of *Asteroschema* and *Ophiocreas* in the two cladograms, also seen in the cladogram of Nethupul et al. (2022), had no impact because all species were in the same clade with the same snakestar body plan. The one exception of “*Asteroschema capense*” in Okanishi and Fujita (2013), presented no problem because of its current placement in genus *Asteromorpha* (Stöhr et al. 2022). The division of six named species of *Astroceras* among three clades by Okanishi and Fujita (2013) and not included by Christodoulou et al. (2019b) indicates that *Astroceras* should be reevaluated by morphologists and phylogeneticists. We accepted the placements per Okanishi and Fujita (2013) for those species of *Astroceras* not shared with Christodoulou et al. (2019b). We treated *Asterostegus sabineae* as “*Asterostegus* sp.” in the cladogram of Okanishi and Fujita (2013). Species of *Trichaster* were not labeled as having branched arms in the cladogram of Okanishi and Fujita (2013), but we treated the genus as being basketstars (Döderlein 1927; Liao and Clark 1995).

Among the Gorgonocephalidae, 34 species were added to the cladograms of Okanishi and Fujita (2013) and Christodoulou et al. (2019b). The genus *Astroclon*, with two species and branched arms, was added based on Okanishi and Fujita (2018). The *Astrodendrum sagaminum* clade of Okanishi and Fujita (2018) presented two problems: Inclusion of “*Gorgonocephalus pustulatus*” was retained upon the consideration that Clark (1916) described the species as *A. pustulatum*; “*Astrodendrum* sp.” in a clade with *Astrophyton muricatum* was ignored for lack of clarifying information. The six species of *Astrodendrum* were nested with all *Gorgonocephalus* based on placement of *G. pustulatum* by Christodoulou et al. (2019b) and Nethupul et al. (2022). Our placement of *A. muricatum* followed Christodoulou et al. (2019b). The distribution of species of the basketstar genus *Astroboa* among four subclades in Okanishi and Fujita (2013) presented no problem with our analysis because all were in a larger clade consisting only of basketstars. Okanishi and Fujita (2013) and Christodoulou et al. (2019b) did not include the following 10 genera, the 12 species of which were, therefore, excluded from our analysis: *Astrocaneum* (2 species), *Astrocnida* (1 species), *Astrocyclus* (2 species), *Astrodictyum* (1 species), *Astrogordius* (1 species), *Astroplegma* (1 species), *Astrospartus* (1 species), and *Schizostella* (1 species) with branched arms; and *Astrozona* (1 species) and *Ophiozeta* (1 species) with simple arms. For family Asteronychidae, seven species were added along with the genera *Astronebris* and *Ophioschiza* (1 species each).

We assumed that simple arms and absence of pedicellariae are ancestral states and that branched arms and presence of pedicellariae are derived states. Sister groups of branched clades were identified, and each pair was assigned a value depending on whether the branched clade (+) or the sister clade (−) had more species. We planned to evaluate results with a one-tailed sign test (Mitter et al. 1988; Farrell et al. 1991; Vamosi and Vamosi 2005) as a conservative nonparametric approach to sister-group comparisons when the number of species in each clade is only approximately known.

## Results

All 12 species of Asteronychidae have five simple arms. Among the 84 species of Euryalidae, 77 species have simple arms, but 9 of them have more than five simple arms (Table 2). Several of the multiarmed Euryalidae are reportedly fissiparous. All seven species of Euryalidae that branch have five arms from which the branches arise. Many (59 of 100 species) of the Gorgonocephalidae have five branched arms. The multiarmed condition is found only in three species: *Astrochlamys sol* (9–12 arms) and *A. timoharai* (11 arms) with simple arms and *Schizostella bifurcata* with seven arms, which branch distally (Table 2). The Euryalidae have, therefore, a greater tendency to have multiple arms, whereas the Gorgonocephalidae tend to have branched arms.

**Table 2:** The multi-armed condition in Euryalida.

Species	Number of arms	Comments	References
**Euryalidae**			
*Asteromorpha koehleri*	6 or 5–7	Fissiparous	Mortensen (1933), O'Hara (2017)
*Asteromorpha tenax*	7		Baker (1980), O'Hara (2017)
*Asteroschema bidwillae*	3–8		McKnight (2000)
*Asteroschema wrighti*	6		McKnight (2000)
*Astroceras annulatum*	6	Fissiparous	Mortensen (1933)
*Astroceras kermadecensis*	5–8		Baker (1980), McKnight (2000)
*Astroceras nodosum*	6–7	Fissiparous	Mortensen (1933)
*Astroceras pleiades*	7		Baker (1980), O'Hara (2017)
*Astrocharis ijimai*	4–6	Fissiparous	Matsumoto (1915), Okanishi and Fujita (2011)
**Euryalidae exceptional cases**			
*Asteroschema oligactes*	6	1 specimen	Verrill (1899)
*Astrobrachion adhaerens*	6	6-armed holotype with 5 jaws	Studer (1885)
*Astroceras pergamenum*	5–7	Misidentified *A. annulatum*	Clark (1911), Mortensen (1933)
*Astrocharis virgo*	5–6	6-armed syntype is *A. ijimai*	Koehler (1904), Okanishi and Fujita (2011)
**Gorgonocephalidae**			
*Astrochlamys sol*	9–12		O'Hara (2017), USNM 1104840*
*Astrochlamys timoharai*	11		Okanishi and Mah (2020)
*Schizostella bifurcata*	7	Fissiparous	Hendler et al. (1995)
**Gorgonocephalidae exceptional cases**			
*Astrosierra microconus*	5–6	6 only in holotype	Clark (1914), Baker (1980)

*Photograph at https://collections.nmnh.si.edu/search/iz/?ark=ark:/65665/374da779cf1c748f89bb8d405e4f4cdfc, visited on April 25, 2022.

**Fig. 3:**
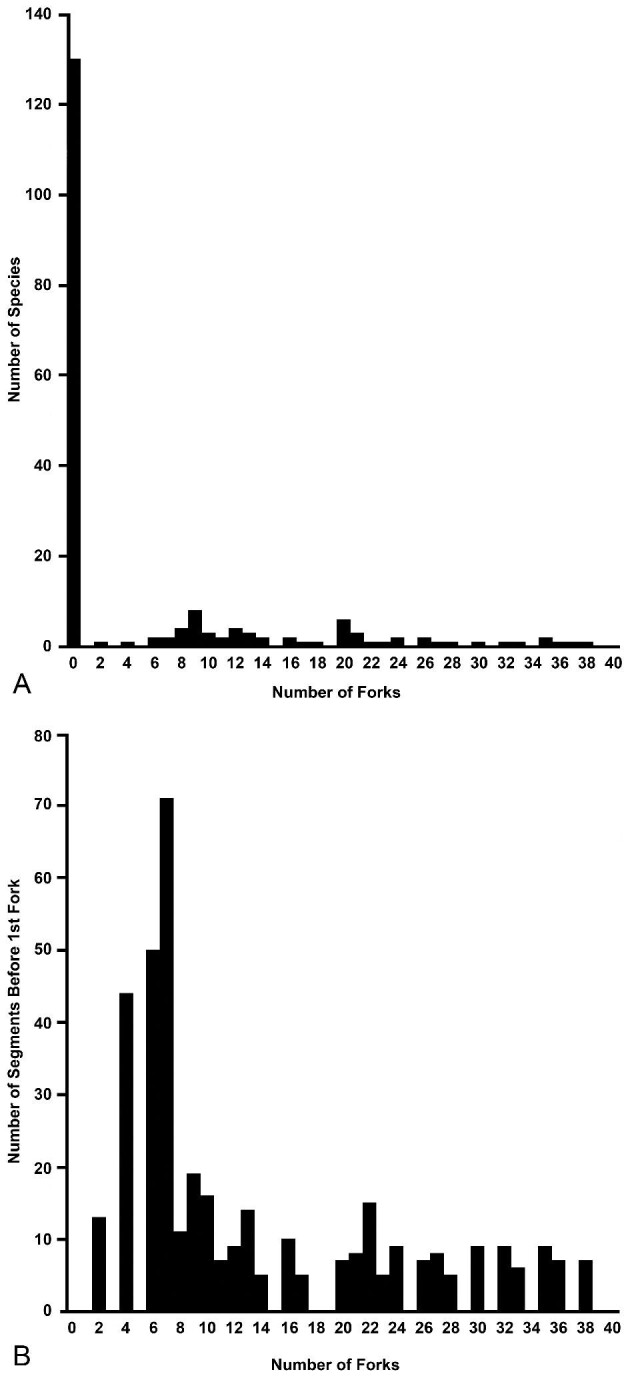
Forking in the Euryalida. A. Maximal number of forks per arm in 191 of 196 species of the Euryalida. Counts were not found in consulted literature for 1 species of Euryalidae and 4 species of Gorgonocephalidae. If the source reported “at least *n* forks,” the count was recorded as “*n* + 1.” B. Number of segments on the arm before the first fork in 51 of 66 species of basketstar for which data were available in the literature. Maximal numbers reported in the literature were used. For species with the same maximal number of forks in the arm, the maximal numbers of segments for the species were averaged.

The majority (130 species) of the Euryalida are snakestars (zero forks; Fig. 3A). Among basketstars (66 species in the Euryalidae and Gorgonocephalidae), the trend is for a high number of branches along the main axis of each arm. In general, the first fork occurs within, at, or just beyond the disc margin (basal branching; Fig. 1A), and the number of segments averages fewer than 10 segments for basketstars with more than 8 forks in an arm (Fig. 3B). In contrast to species with basal branching, all six species with seven or fewer forks in the arm have the first fork well beyond the disc (11–80 segments to first fork) and closer to the distal tip of the arm (distal branching; Figs. 1B and 3B). Among the 15 species with 8–10 forks, 40% (six species) have distal branching (10–48 segments to first fork). All species with 11 or more forks have basal branching. There are, therefore, few (21) species of basketstar with minimally forked arms, and most of those species (12) have distal branching. The branched condition is, therefore, bimodal: 130 species have no forks, and 54 species with basal branching have eight or more forks. The intermediate range of two to seven forks is occupied only by the few species with distal branching.

The seven species of basketstar in the Euryalidae are in two clades (Fig. 2): five species in *Euryale* and *Trichaster*; and two in *Sthenocephalus*. Compared to their sister groups, the branched clades are equally diverse (*Euryale*–*Trichaster* clade versus *Asteromorpha*–*Astroceras kermadecensis* clade) or much less diverse (*Sthenocephalus* clade versus *Asterostegus–Astroceras nodosum/spinigerum* clade) (Table 3). There is no evidence that branching of arms has been an adaptive zone within the Euryalidae.

**Table 3:** Sister-group comparisons of basketstar clades and their sister snakestar clades.

Branched clade	Number of species	Sister clade	Number of species	Outcome
**Euryalidae**				
*Sthenocephalus*	2	*Asterostegus *+ 2 *Astroceras* spp.	5	−
*Trichaster *+ *Euryale*	5	*Asteromorpha *+ 1 *Astroceras* sp.	5	0
**Gorgonocephalidae**				
*Astroclon*	2	*Astrothrombus*	4	−
*Astrodendrum *+ *Gorgonocephalus*	16	*Astrochele + Astrochlamys*	6	+
*Astrophyton*	1	*Asteroporpa + Astrogomphus*	13	−
*Astracme + Astroboa* + *Astrochalcis* + *Astrocladus* + *Astroglymma* + *Astrosierra* + *Conocladus* + *Ophiocrene*	30	*Asteroporpa *+ *Astrogomphus* + [Astrophyton*]	14	+
				

*Branched arms.

Outcome: +, branched clade more speciose than sister clade; −, branched clade less speciose than sister clade; 0, both clades with the same number of species.

Excepting 10 species of basketstar that cannot yet be place in the cladogram, four clades of Gorgonocephalidae have branched arms (Fig. 2, Table 3): 2 species of *Astroclon*; 6 species of *Astrodendrum* with 10 species of *Gorgonocephalus; A. muricatum*; and 30 species in a clade of the genera *Astracme, Astroboa, Astrochalcis, Astrocladus, Astroglymma, Astrosierra, Conocladus*, and *Ophiocrene*. The first clade of gorgonocephalid basketstars, *Astroclon*, with two distally branching species, is less diverse than its sister clade of snakestars, *Astrothrombus*, with four species. On the other hand, the second clade of basketstars, *Astrodendrum* and *Gorgonocephalus*, with 16 species, is much more diverse than its sister clade *Astrochele* and *Astrochlamys*, with 6 species. Third, the sole basketstar *A.muricatum* is sister to the 13 snakestar species of *Asteroporpa* and *Astrogomphus.* The fourth clade of basketstars—*Astracme* and its seven sister genera—has 30 species compared to its sister clade of 14 species, a mixed clade of 13 snakestars (*Asteroporpa-Astrogomphus*) and the basketstar *A. muricatum*. Unfortunately, a one-tailed sign test cannot be applied to sister-group comparisons with *n* < 5.

Whereas branching evolved four to six times in the Euryalida, the distribution of pedicellariae in the order is simpler: they evolved once and that only in the Gorgonocephalidae. This large family of 100 species is more diverse than its sister clade, the Euryalidae (86 species).

## Discussion

With about 2100 living species in 194 genera and 29 families (Stöhr et al. 2012, 2022; O'Hara 2017), the class Ophiuroidea is the most diverse group of extant echinoderms. Only 66 species (3% of ophiuroids) have branched rays, and 100 species have pedicellariae. All species with these two characters are members of the order Euryalida (196 species in 48 genera in 3 families). The distribution of these characters is unequal among and within the three euryalidan families: the Asteronychidae (12 species in 4 genera) are snakestars without pedicellariae; the Euryalidae (84 species in 11 genera) have only 7 species of basketstar, and no species has pedicellariae; and the Gorgonocephalidae (100 species in 33 genera) include 59 species of basketstar, and all members of the family bear pedicellariae. The order Ophiurida, sister group to the Euryalida (O'Hara et al. 2018), by contrast have more than twice the species diversity (404 species in 46 genera in 5 families; Stöhr et al. 2022). Branching evolved twice in the Euryalidae and up to four times in the Gorgonocephalidae (Fig. 2). With the phylogenetic position of 10 genera of gorgonocephalid unknown, branching could have arisen independently even more times. Pedicellariae, on the other hand, evolved only once; all gorgonocephalids and only this family have this character (Turner et al. 2021).

Our focus has been the phylogenetic distribution of two characters of branching: the position of the first fork and the degree of proliferation of branching. All 12 species with the first fork well beyond the disk have 10 or fewer forks along a main axis of an arm. This condition—distal branching—predominates in basketstars of the Euryalidae (5 of 7 species), a family composed mostly of snakestars; it is rare in the Gorgonocephalidae (only in 7 of 59 species of basketstar), which has many more basketstar than snakestar species. Branching seems not to have been a key character leading to diversification in the Euryalidae; this conclusion is supported by the weak sister-group comparisons of the two branching clades (Tables 3, 4). Also among the Gorgonocephalidae, distal branching and presence of few forks have not led to high diversification (Table 4), although *Astrosierra* (nested within a large clade with basal branching) is more speciose than *Conocladus*, its sister group (Okanishi and Fujita 2013; Christodoulou et al. 2019b). But the two clades with basal branching and high numbers (8–38) of forks are more speciose than their sister clades of snakestars. Basal branching probably gives biomechanically stronger support for further branching of the arms because the first fork and sometimes the second or third forks (*Astroboa nuda, Astrocaneum herrerai, Astrocyclus somaliensis, G. eucnemis*; Clark 1919; Döderlein 1927; Baker et al. 2018) are integral with the disc, at least in mature animals with large discs. Basal branching also occurs in crinoid rays, in which the first axillary (fork brachial) is usually part of the calyx (Clark 1921; Lawrence 1987, 2012). Incorporation of basal forks into the central body (disc or calyx) of these two diverse taxa indicates a need for a stable basis for branching of arms.

**Table 4:** Sister-group comparisons of basketstars with distal branching and their sister clades.

Genus with distal branching	Number of species	Sister clade (S, snakestar; B, basal branching)	Number of species	Outcome
**Euryalidae**				
*Sthenocephalus*	2	*Asterostegus* (S) + 2 *Astroceras* spp. (S)	5	−
*Trichaster*	3	*Euryale* (B)	2	+
**Gorgonocephalidae**				
*Astroclon*	2	*Astrothrombus* (S)	4	−
*Astrosierra*	3	*Conocladus* (B)	1	+
Astrocnida*	1	Unknown		
Schizostella*	1	Unknown		

*Genera not included in analyses by Okanishi and Fujita (2013, 2018) and Christodoulou et al. (2019b).

Outcome: +, clade with distal branching more speciose than sister clade; −, clade with distal branching less speciose than sister clade.

Branching has evolved in the Euryalida four to six times, within the Gorgonocephalidae only two to four of those times based on current knowledge. Sister-group analysis in the literature has been applied to other groups with many more species and greater numbers of independent evolution of characters of interest (e.g., Mitter et al. 1988, for phytophagy in insect diversification; Farrell et al. 1991, for latex and resin canals in angiosperm diversification). Only four paired comparisons within Gorgonocephalidae were available for sister-group comparisons, and that number is too low for application of a one-tailed sign test. These four comparisons cannot be combined with the two in the Euryalidae because of the additional and potentially confounding character of pedicellariae in the Gorgonocephalidae. That is, branching and pedicellariae might not be independent variables; “branching + pedicellariae” might have a different influence on diversification rate than either trait has alone (Jablonski 2008). Our analysis is, therefore, only by inspection of data and lacks the support of statistical application of the sign test because of low replication; we presently know that pedicellariae have evolved once in ophiuroids and that branching in gorgonocephalids has evolved only two to four times. On the other hand, there were (and remain) no genetic data on 8 genera of gorgonocephalid basketstar with their 10 species for their inclusion in our combined cladogram based on those of Okanishi and Fujita (2013, 2018) and Christodoulou et al. (2019b). Their cladograms also did not include 2 genera with 2 species of gorgonocephalid snakestar. Future work with greater taxon sampling might reveal a pattern of diversification with greater assurance by application of the non-parametric one-tailed sign test.

Branching of rays occurs in extant echinoderms only in the two major suspension-feeding groups: crinoids and euryalidan ophiuroids (Emson 1990; Emson et al. 1991; Lawrence 1987, 2012; Messing et al. 2021). Branching probably confers a selective advantage for suspension feeding, but it also imposes constraints on benthic locomotion, as Lawrence (2012) argued. These two taxa have limited mobility, and neither includes species with burrowing lifestyles. But, if branching is potentially advantageous for suspension feeding, then why has it not led to high diversification in the Euryalidae as it has in the Gorgonocephalidae? The explanation might be found in the replacement of downstream podial-mucus capture of prey in the Euryalidae (like that of crinoids; Lawrence 1987) with upstream pedicellarial capture of prey in the Gorgonocephalidae (Wolfe 1978; Emson 1990; Emson et al. 1991). Were it not for pedicellariae, branching might not have led to high diversification in gorgonocephalid clades with branched arms.

The rise of the Euryalida in the early Cenozoic (O'Hara et al. 2018) is probably attributable to their adaptations to a new ecospace of suspension feeding on reef outcrops and colonial anthozoans and sponges (Bambach 1985; Emson 1990; O'Hara 2017). Although new ecospace for ophiuroids, euryalidans could have been in competition with comatulid crinoids (Messing et al. 2021) in reef systems. Differing prey sizes might, however, have allowed for resource partitioning (Meyer 1982): Euryalidans feed on larger zooplankton (copepods, euphausiids, polychaetes, chaetognaths, larvaceans, larval fish, and others; Fedotov 1926; Wolfe 1978; Dearborn et al. 1986; Emson et al. 1991) than do comatulids, which also capture phytoplankton (Messing et al. 2017). Members of the family Euryalidae (predominately snakestars) have retained the typical down-stream podial prey-capture mechanism of suspension- and detrital-feeding relatives, and branching seems not to have led to species diversification. Among the Gorgonocephalidae (predominately basketstars), on the other hand, the key innovation of prey capture by gorgonocephalous pedicellariae seems to have conferred a great advantage to branching of the rays; clades with basal branching consistently exceed their sister groups in species diversity. In his treatment of diversity replacement, Bambach (1985) pointed to the replacement of fossil stenurid and oegophiurid ophiuroids by “phrynophiurids” (euryalidans) and ophiurids with key innovations related to mobility and feeding. With their occupation of a limited ecospace (epizoic suspension feeders on larger zooplankton in reef systems) by the Euryalida, gorgonocephalids with pedicellarial prey capture might, in future eons, largely or fully replace other euryalids with podial prey capture; and gorgonocephalid basketstars with a greater prey-capture network might eventually replace gorgonocephalid snakestars.

## Data Availability

A data set of euryalidan characters extracted from literature for this study is available at https://repository.fit.edu/oems_faculty/36/.
